# CO-CREATING RESOURCES TO RAISE AWARENESS OF RISKS OF GOING MISSING AMONG PERSONS WITH DEMENTIA

**DOI:** 10.1093/geroni/igae098.3308

**Published:** 2024-12-31

**Authors:** Christine Daum, Vanessa Vahedi, Isabella Chawrun, Noelannah Neubauer, Emily Rutledge, Adebusola Adekoya, Antonio Miguel-Cruz, Lili Liu

**Affiliations:** University of Waterloo, Waterloo, Ontario, Canada; University of Waterloo, Waterloo, Ontario, Canada; University of Waterloo, Waterloo, Ontario, Canada; University of Waterloo, Waterloo, Ontario, Canada; University of Waterloo, Waterloo, Ontario, Canada; University of Waterloo, Waterloo, Ontario, Canada; University of Alberta, Edmonton, Alberta, Canada; University of Waterloo, Waterloo, Ontario, Canada

## Abstract

Persons living with dementia are at risk of getting lost and going missing due to wayfinding challenges. Yet, there are few accessible resources available that raise awareness of the risks of going missing and strategies to manage these risks. The purpose of this project was to develop engaging and informative learning resources for first responders, search and rescue personnel, persons living with dementia, care partners, and health and social care providers. Co-design and co-production approaches guided the development of these resources and were informed by reviews of the academic and grey literature, dialogue with persons with lived experiences, and content analysis of missing person cases. We produced three sets of resources: 1) nine dementia-friendly first responder videos for police and search and rescue personnel to enhance their awareness of dementia and risks of going missing; 2) a toolkit of strategies and resources for persons living with dementia and care partners to reduce the risks associated with getting lost and going missing; 3) eight personas and scenarios that depict contextual factors within missing events that can be utilized by service providers and the public in their interactions with persons living with dementia and care partners. All resources were reviewed by participants through interviews, focus groups, and written feedback to identify essential content and ensure accuracy, relevance, and comprehensibility. These sets of learning resources can elevate awareness and offer practical strategies to support persons living with dementia, care partners, first responders, and service providers.

